# Cardiac magnetic resonance imaging and cardiac scintigraphy in the diagnosis of cardiac amyloidosis: A meta-analysis of 4866 patients

**DOI:** 10.1016/j.jmccpl.2025.100489

**Published:** 2025-10-17

**Authors:** Mahmoud Balata, Abdelrahman M.Attia, Mohamed Ibrahim Gbreel, Mamdouh Elsmaan, Marwa Hassan, Paul Rapeanu, Milka Marinova, Marc Ulrich Becher, Islam Ebeid, Jasmin Ortak, Hüseyin Ince

**Affiliations:** a Department of Internal Medicine and Cardiology, University Hospital Rostock, Ernst-Heydemann-Straße 6, 18057, Rostock, Germany; b Karsh Division of Gastroenterology and Hepatology, Comprehensive Transplant Center, Cedars-Sinai Medical Center, Los Angeles, CA, USA; c Faculty of Medicine, 6th of October University, Giza, Egypt; d Guy's and St Thomas' NHS Foundation Trust, Westminster Bridge Rd, London, SE1 7EH, United Kingdom; e Immunology Department, Theodor Bilharz Research Institute, Giza, 12411, Egypt; f Department of Cardiology, Marienhospital Aachen, Zeise 4, 52066, Aachen, Germany; g Department of Nuclear Medicine, University Hospital Bonn, Venusberg-Campus 1, 53127, Bonn, Germany; h Department of Internal Medicine II, Städtisches Klinikum Solingen, Gotenstraße 1, 42653, Solingen, Germany; i National institute of diabetes and endocrinology, 16 Al Kasr Al Aini, El Sayeda Zeinab, Cairo Governorate, 4260010, Egypt

**Keywords:** Cardiac amyloidosis, Cardiac scintigraphy, CMR imaging, Non-invasive diagnosis, 99mTc-HMDP, 99mTc-DPD

## Abstract

**Introduction:**

Cardiac amyloidosis (CA) impacts about 20 % of elderly heart failure patients, leading to myocardial dysfunction and life-threatening risks. However, it often remains undetected due to the significant risks associated with invasive biopsies. This highlights the critical need for safer and accurate non-invasive diagnostic techniques.

**Aim:**

To compare the diagnostic value of Cardiac Magnetic Resonance (CMR) imaging and Cardiac Scintigraphy Imaging in the diagnosis of CA.

**Methods:**

A comprehensive literature search across PubMed, Scopus, Web of Science, and Cochrane databases yielded studies that utilized CMR or cardiac scintigraphy for diagnosing CA. QUADAS-2 was employed for quality assessment.

**Results:**

From 7117 records, 35 studies involving 4866 patients were analyzed. Cardiac scintigraphy demonstrated higher sensitivity and specificity across different radiotracers, with 99mTc-HMDP showing the highest specificity (1.00, 95 % CI: 0.93–1.00) and 99mTc-DPD the highest sensitivity (0.93, 95 % CI: 0.89–0.95). CMR imaging showed variable diagnostic accuracy with a sensitivity of 0.83 (95 % CI: 0.81–0.85) and a lower specificity of only 0.53 (95 % CI: 0.50–0.56).

**Conclusion:**

Cardiac scintigraphy, particularly with 99mTc-HMDP, offers superior diagnostic accuracy for CA compared to CMR imaging. Controlled, randomized, prospective studies directly comparing these non-invasive techniques are essential to validate these findings.

## Highlights

• Cardiac scintigraphy with 99mTc-HMDP enables accurate and reliable diagnosis of cardiac amyloidosis.
• Different radiotracers used in Cardiac Scintigraphy exhibit varying levels of diagnostic accuracy.
• CMR is useful for cardiac imaging but less specific than scintigraphy in distinguishing amyloidosis.
• Diagnostic method selection should be individualized to each patient's clinical scenario.

## Graphical abstract

**Figure:**
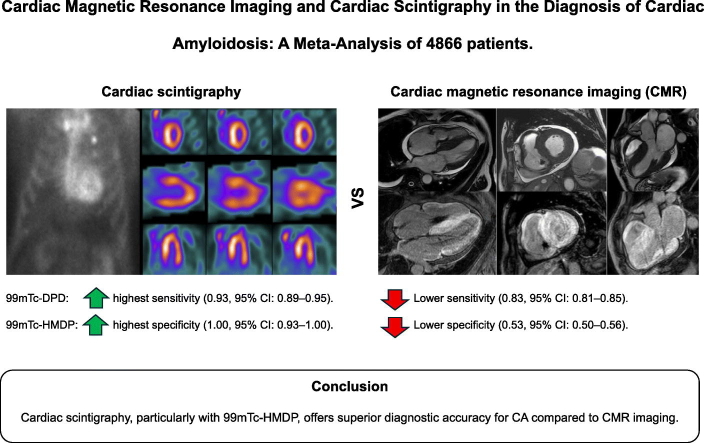

## Key Learning Points

**What is already known:**

Cardiac amyloidosis (CA) is significantly prevalent among elderly heart failure patients, often undetected due to the risks of invasive biopsies, underlining the need for accurate non-invasive diagnostic methods.

**What this study adds:**

This study provides a detailed comparison between Cardiac Magnetic Resonance (CMR) imaging and Cardiac Scintigraphy, demonstrating that scintigraphy, especially with 99mTc-HMDP, is more accurate than CMR in diagnosing CA.

## 1 Introduction

Cardiac amyloidosis (CA) is a myocardial disease characterized by abnormal extracellular deposition of amyloid fibrils leading to progressive cardiac dysfunction and potentially life-threatening outcomes [1,2]. Despite its serious prognosis, CA's subtle symptomatology and diagnostic complexities often result in underdiagnosis [3]. Emerging evidence suggests that CA is underreported, potentially affecting up to 20 % of heart failure patients aged 65 years and older [4]. This underestimation highlights the critical need for precise and accessible diagnostic methods.

The endomyocardial biopsy, while the gold standard for diagnosing CA, is invasive and carries significant risks, rendering it less suitable for many patients [5]. This underscores the importance of safer, non-invasive diagnostic alternatives for this vulnerable patient group prone to comorbidities. Therefore, non-invasive imaging modalities like Cardiac Magnetic Resonance (CMR) imaging and Cardiac Scintigraphy Imaging have emerged as pivotal in the diagnostic algorithm of CA [6]. CMR imaging provides comprehensive visualization of cardiac structure and function, facilitating the identification of amyloid deposits [7]. Conversely, cardiac scintigraphy employs Technetium-labelled radiotracers for the detection of amyloid deposits within the myocardium [8].

The distinct advantages and inherent limitations of CMR and Cardiac Scintigraphy necessitate a detailed comparison of their diagnostic capabilities. This meta-analysis aims to compare these non-invasive modalities in diagnosing CA to identify the most effective diagnostic approach, ultimately facilitating detection, enhancing patient care, and reducing reliance on invasive diagnostic procedures.

## 2 Methods

### 2.1 Search strategy

A meticulous search was conducted in the PubMed, Scopus, Web of Science, and Cochrane databases up through July 2023, focusing on English-language literature related to CA. This search strategy included the utilization of Medical Subject Headings (MeSH) terms and specific keywords associated with the diagnosis of CA, such as CMR and cardiac scintigraphy as well as reference diagnostic tests like endomyocardial or extracardiac biopsy. To broaden the literature review's scope, manual screening of references from relevant studies and review articles was performed. This diligent process identified 4649 records. Subsequently, two independent reviewers (MB and MH) carefully screened the titles and abstracts for relevance before reviewing the full texts in detail to determine the eligibility of the studies for inclusion.

### 2.2 Inclusion and exclusion criteria

Adhering to the PRISMA guidelines [9,10], the criteria for inclusion were defined as studies assessing patients with CA who were investigated and diagnosed using either CMR or cardiac scintigraphy, compared against a reference test. This reference involved histological verification of tissue samples sourced from either an extracardiac organ or through an endomyocardial biopsy. Any studies that did not provide sufficient data to enable the calculation of sensitivity or specificity for CMR or cardiac scintigraphy were excluded.

### 2.3 Quality assessment

Two independent reviewers utilized QUADAS-2 [11,12]: a revised tool for the quality assessment of diagnostic accuracy studies to rigorously evaluate the quality of the included studies. Implementing the QUADAS-2 tool involves four essential steps: defining the review questions, adapting the tool and developing specific instructions for the review, creating a detailed flow diagram to outline the study selection process, and assessing the risk of bias and the applicability of the study findings. This systematic approach ensures a comprehensive and standardized assessment of the methodological quality and relevance of the diagnostic accuracy studies included in the review.

### 2.4 Data extraction and statistical analysis

Meta-Disc (version 1.4) was employed for data analysis, with a *P*-value of less than 0.05 considered statistically significant. Anticipating heterogeneity between studies due to clinical and methodological differences, a random-effects model was utilized for the analysis. The sensitivity, specificity, diagnostic odds ratio (dOR), positive likelihood ratio (PLR), negative likelihood ratio (NLR), and the area under the Receiver Operating Characteristic curve (ROC) along with their 95 % confidence intervals (CIs) were derived from the counts of true positives, false positives, false negatives, and true negatives.

## 3 Results

**Fig. 1:**
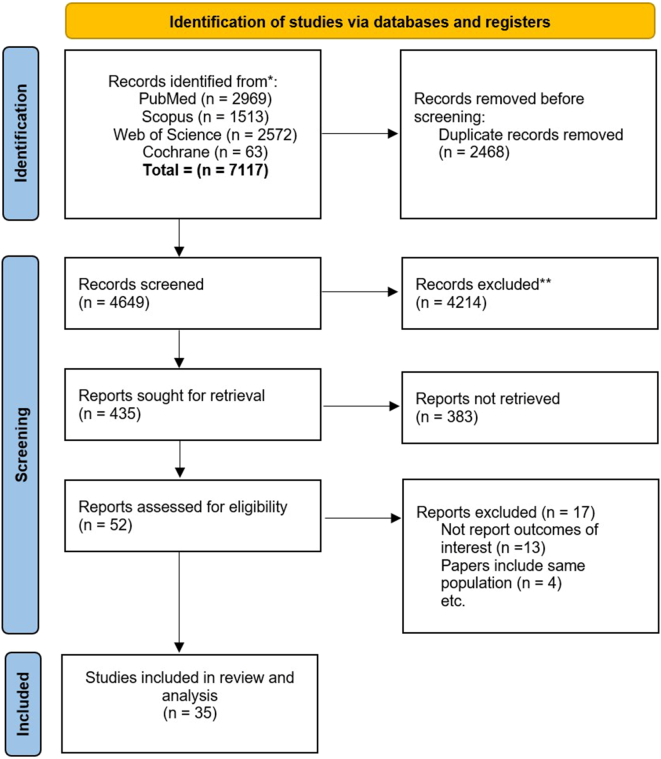
PRISMA flow diagram of study selection.

A total of 7117 records were initially identified for inclusion in the study. Following the removal of duplicate entries, 4649 potentially relevant studies remained. Subsequent screening based on title and abstract resulted in the exclusion of 4214 studies deemed irrelevant to the research objectives. Further refinement through full-text screening narrowed down the selection to 52 eligible studies. However, data extraction was not feasible for 17 of these studies, leaving a final set of 35 articles for inclusion in the analysis (Fig. 1).

Sixteen articles concentrated on cardiac scintigraphy, including five prospective cohort studies and 11 retrospective cohort studies. These studies collectively involved 3206 patients [5,[13], [14], [15], [16], [17], [18], [19], [20], [21], [22], [23], [24], [25], [26], [27]]. The CMR studies included 19 articles, consisting of 11 prospective cohort studies and eight retrospective studies, among which were six cohort and two cross-sectional studies, covering 1660 patients [[28], [29], [30], [31], [32], [33], [34], [35], [36], [37], [38], [39], [40], [41], [42], [43], [44], [45], [46]]. Baseline patient characteristics of the included cardiac scintigraphy studies and CMR studies are detailed in Supplemental Tables 1 and 2, respectively.

### 3.1 Quality assessment

Among the 35 studies included, nine were assessed as high-risk in the domain of patient selection, two in the index test, and 13 exhibited high-risk at the flow and timing stage. Most studies were deemed low risk. Nonetheless, six studies [5,15,20,22,24,41] were classified as having an unclear risk of bias due to several factors, such as indeterminate cohort selection methods or the potential inclusion of duplicate patients (Supplemental Tables 3).

### 3.2 The diagnostic performance of cardiac scintigraphy and CMR for diagnosing CA

#### 3.2.1 99mTc-DPD (Technetium-99 m-labelled 3,3-diphosphono-1,2-propanodicarboxylic acid scintigraphy) imaging efficacy

**Fig. 2:**
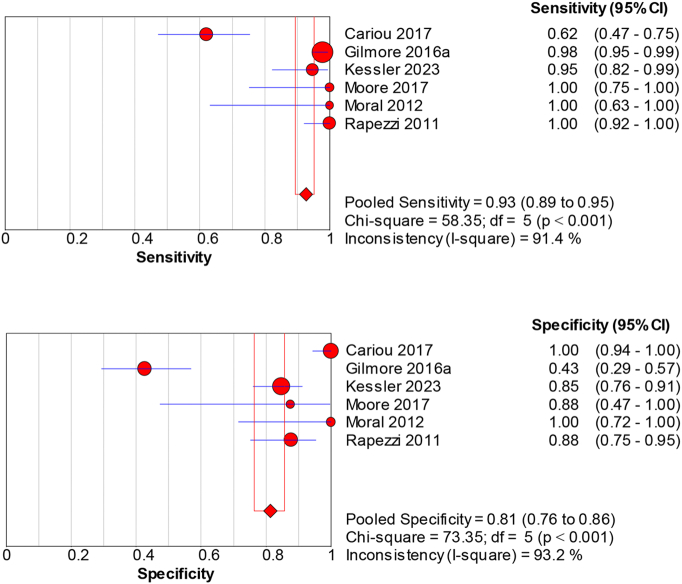
Sensitivity and specificity of 99mTc-DPD for diagnosing cardiac amyloidosis. Pooled sensitivity = 0.93 (95 % CI: 0.89–0.95) and specificity = 0.81 (95 % CI: 0.76–0.86) across cohorts. Error bars show 95 % CI. Heterogeneity: sensitivity *p* < 0.001, I^2^ = 91.4 %; specificity p < 0.001, I^2^ = 93.2 %. Model: random-effects. (see Supplementary Table 1 for study-level counts).

For CA, 99mTc-DPD imaging achieves a sensitivity of 0.93 (95 % CI: 0.89–0.95) and specificity of 0.81 (95 % CI: 0.76–0.86) (Fig. 2). Its diagnostic efficacy is underscored by a dOR of 85.34 (95 % CI: 35.38–205.86), a PLR of 6.66 (95 % CI: 2.37–18.67), and a NLR of 0.068 (95 % CI: 0.01–0.32), despite significant study variability (sensitivity *p* < 0.001, I^2 = 91.4 %; specificity p < 0.001, I^2 = 93.2 %) (Supplemental Fig. 1).

#### 3.2.2 99mTc-HMDP (Technetium-99 m-labelled hydroxymethylene- diphosphonate scintigraphy) imaging efficacy

**Fig. 3:**
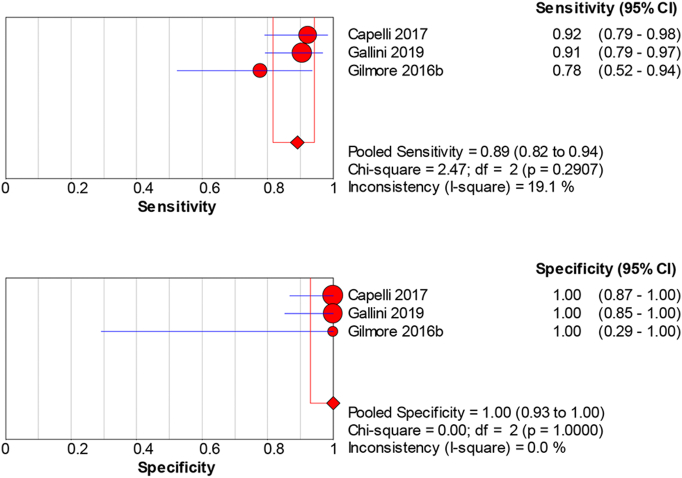
Sensitivity and specificity of 99mTc-HMDP for diagnosing cardiac amyloidosis. Pooled sensitivity = 0.89 (95 % CI: 0.82–0.94) and specificity = 1.00 (95 % CI: 0.93–1.00) across cohorts. Error bars: 95 % CI. Heterogeneity: sensitivity *p* = 0.29, I^2^ = 19.1 %; specificity *p* = 1.00, I^2^ = 0 %. Model: random-effects. (see Supplementary Table 1 for study-level counts).

99mTc-HMDP imaging exhibits sensitivity at 0.89 (95 % CI: 0.82–0.94) and a specificity of 1.00 (95 % CI: 0.93–1.00) (Fig. 3), with minimal variability in findings, indicating consistent performance (sensitivity *p* = 0.29, I^2 = 19.1 %; specificity *p* = 1.00, I^2 = 0 %). An exceptionally high dOR of 183.46 (95 %CI: 25.78–1305.32), a PLR of 22.39 (95 % CI: 4.72–106.19), and an NLR of 0.14 (95 % CI: 0.06–0.28) highlight its precision in diagnosis (Supplemental Fig. 2).

#### 3.2.3 99mTc-PYP (99mTechnetium-pyrophosphate scintigraphy) imaging efficacy

**Fig. 4:**
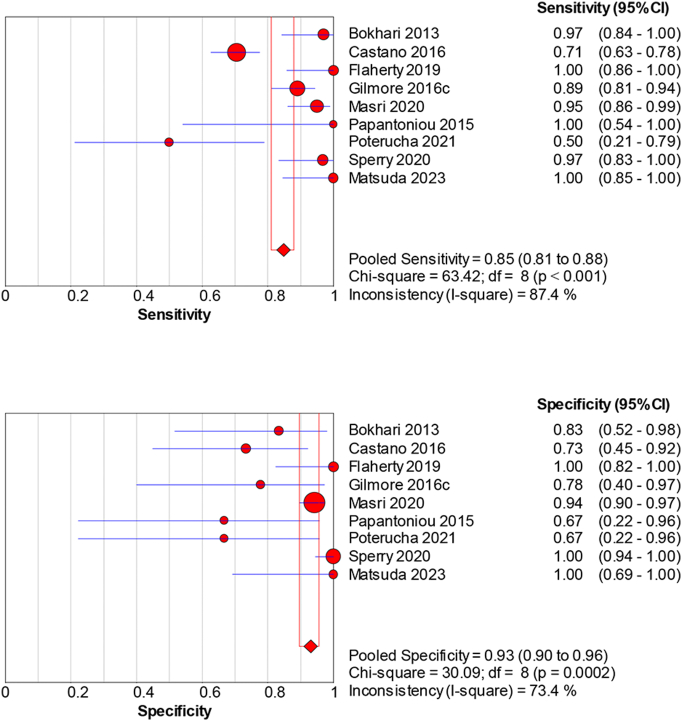
Sensitivity and specificity of 99mTc-PYP for diagnosing cardiac amyloidosis. Pooled sensitivity = 0.85 (95 % CI: 0.81–0.88) and specificity = 0.93 (95 % CI: 0.90–0.96) across cohorts. Error bars: 95 % CI. Heterogeneity: sensitivity *p* ≤ 0.001, I^2^ = 87.4 %; specificity *p* = 0.0002, I^2^ = 73.4 %. Model: random-effects. (see Supplementary Table 1 for study-level counts).

99mTc-PYP imaging shows a sensitivity of 0.85 (95 % CI: 0.81–0.88) and a specificity of 0.93 (95 % CI: 0.90–0.96) (Fig. 4), albeit with notable study variability (sensitivity *p* ≤ 0.001, I^2 = 87.4 %; specificity *p* = 0.0002, I^2 = 73.4 %). A dOR of 79.1 (95 %CI: 15.62–400.65), a PLR of 6.45 (95 % CI: 2.79–14.92), and an NLR of 0.10 (95 % CI: 0.03–0.31) underscore its effectiveness (Supplemental Fig. 3).

#### 3.2.4 General cardiac scintigraphy imaging efficacy

**Fig. 5:**
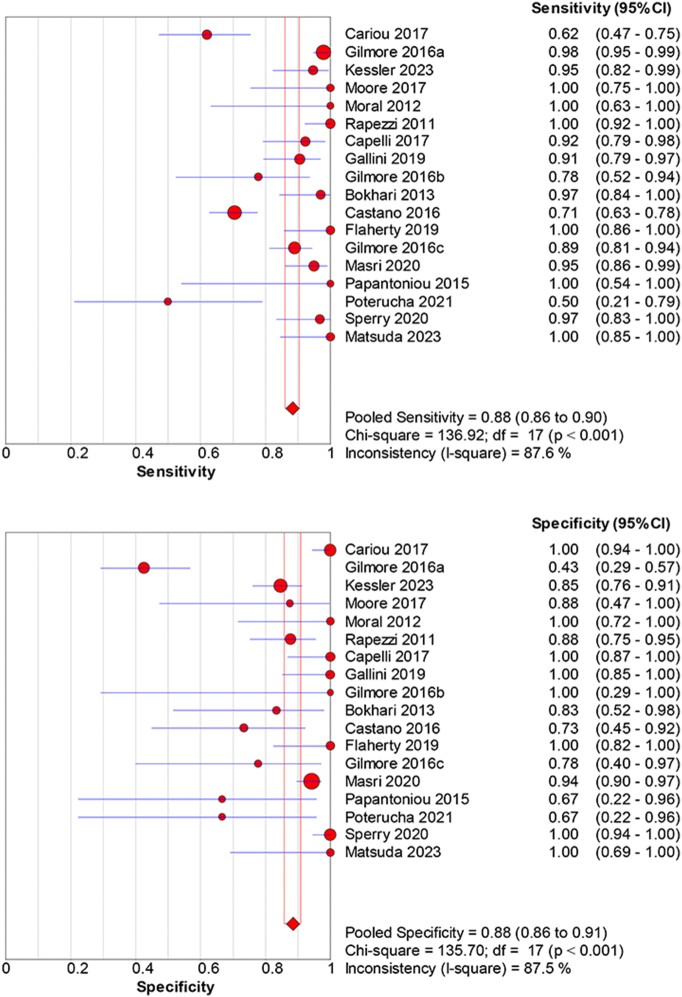
Pooled diagnostic accuracy of cardiac scintigraphy (all tracers combined). Pooled sensitivity = 0.88 (95 % CI: 0.86–0.90) and specificity = 0.88 (95 % CI: 0.85–0.91) across cohorts. Error bars: 95 % CI. Heterogeneity: sensitivity and specificity p < 0.001, I^2^ = 87.5 %. Model: random-effects. (see Supplementary Table 1 for study-level counts).

A collective analysis of all types of cardiac scintigraphy imaging types reveals an average sensitivity and specificity of 0.88 (95 % CI: 0.86–0.90) and 0.88 (95 % CI: 0.85–0.91) (Fig. 5), with significant variability (sensitivity and specificity *p* < 0.001, I^2 = 87.5 %). The diagnostic value is reinforced by a dOR of 98.68 (95 %CI: 38.9–250.4), PLR of 7.78 (95 % CI: 3.93–15.39), and NLR of 0.11 (95 % CI: 0.05–0.19) (Supplemental Fig. 4).

#### 3.2.5 CMR imaging efficacy

**Fig. 6:**
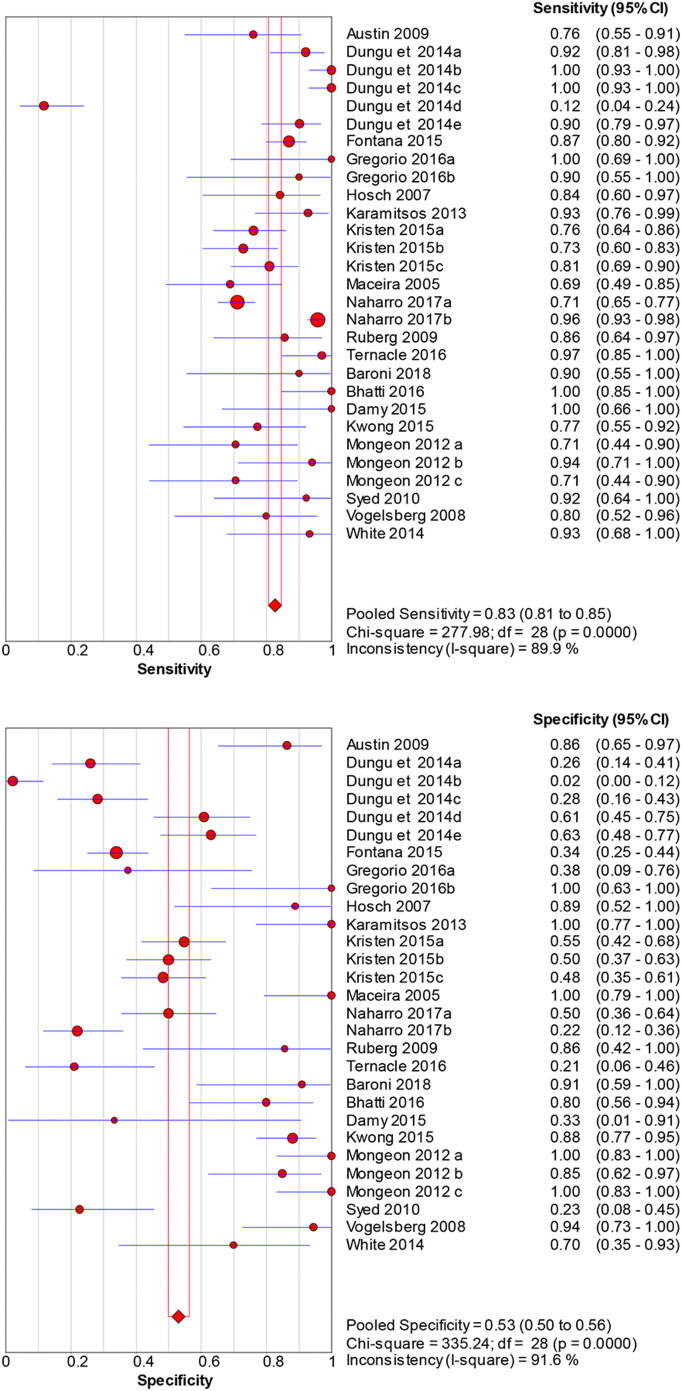
Diagnostic accuracy of cardiac MRI (CMR) for cardiac amyloidosis. Pooled sensitivity = 0.83 (95 % CI: 0.81–0.85) and specificity = 0.53 (95 % CI: 0.50–0.56) across cohorts and *n* = 1660 patients (CMR studies). Error bars: 95 % CI. Heterogeneity: sensitivity and specificity p < 0.001, I^2^ > 89 %. Model: random-effects. (see Supplementary Table 1 for study-level counts).

CMR demonstrates diagnostic accuracy with a sensitivity of 0.83 (95 % CI: 0.81–0.85) and specificity of 0.53 (95 % CI: 0.50–0.56) (Fig. 6), despite considerable variability (sensitivity and specificity p < 0.001, I^2 > 89 %). Its diagnostic effectiveness is emphasized by a dOR of 11.29 (95 % CI: 6.42–19.84), a PLR of 1.98 (95 % CI: 1.55–2.52), and a NLR of 0.25 (95 % CI: 0.18–0.37) (Supplemental Fig. 5).

## 4 Discussion

This study comprehensively evaluates the diagnostic performance of cardiac scintigraphy using Technetium (Tc)-labelled radiotracers (99mTc-DPD, 99mTc-HMDP, 99mTc-PYP) against CMR imaging for identifying CA. Our analysis indicates that 99mTc-DPD and 99mTc-HMDP imaging have the highest sensitivity, demonstrating their superior ability to accurately identify CA in patients. Specifically, 99mTc-HMDP stands out for its specificity, offering the highest accuracy in correctly identifying individuals without the condition, closely followed by 99mTc-PYP. Overall, our findings support the use of cardiac scintigraphy, especially 99mTc-HMDP, as having greater diagnostic accuracy for detecting or excluding CA compared to CMR imaging.

CMR imaging is essential in the diagnostic process of CA, offering detailed insights into cardiac structure, function, and tissue characteristics [7]. However, its effectiveness is challenged in distinguishing CA from conditions with similar presentations, such as hypertrophic cardiomyopathy [48]. To overcome these obstacles, the diagnostic approach incorporates cine CMR, supplemented by late gadolinium enhancement (LGE) to identify amyloid protein infiltration [7]. Moreover, LGE serves as a significant prognostic marker in AL amyloidosis, where transmural LGE is linked to adverse outcomes [28,32,41]. Nevertheless, the utility of LGE in the early stages of detection is limited, and its specificity for CA can be compromised by the presence of extensive fibrosis common in other diseases [28,32,49].

The use of gadolinium-based contrast agents is contraindicated in patients with severe renal failure, a condition relatively prevalent among individuals with amyloidosis [50]. Consequently, T1 and T2 mapping techniques, along with extracellular volume (ECV) measurement, have emerged as essential alternative diagnostic tools. Elevated T1 mapping values and ECV are indicative of amyloid protein accumulation, effectively differentiating CA patients from healthy individuals [41,51]. Moreover, T1 mapping offers a unique capability to differentiate CA from phenotypically similar conditions such as Anderson-Fabry disease, which is characterized by markedly lower T1 times due to fatty infiltration [41].

Despite their benefits, the utility of T1 and T2 mapping is constrained by variability in reference ranges, differences in mapping sequences, magnet field strength, and the complexity of differentiating between interstitial and myocyte signal intensity [52]. This necessitates the establishment of local reference ranges by each imaging facility [53]. Furthermore, the increase in myocardial native T1 times, indicative of cardiac fibrosis in CA, may also manifest in the early stages of chronic kidney disease, complicating the specificity of these measures for CA diagnosis [54].

Cardiac scintigraphy, using Technetium (Tc)-labelled radiotracers derived from phosphate-based bone scanning agents, is a key diagnostic tool for CA [5]. Our meta-analysis examining the diagnostic performance of various radiotracers has identified differences in sensitivities and specificities across studies. Specifically, 99mTc-DPD and 99mTc-HMDP show the highest sensitivity, while 99mTc-HMDP and 99mTc-PYP exhibit the highest specificity. Although the precise mechanisms through which these tracers detect CA remain unclear, the observed variability indicates differences in their affinity for amyloid proteins.

Our findings highlight the superior diagnostic accuracy of cardiac scintigraphy over CMR imaging in both detecting and ruling out CA. Nevertheless, it's important to recognize the diagnostic limitations of this method. Cardiac scintigraphy may yield false positive and false negative results. For example, AL amyloidosis patients might display positive uptake similar to TTR amyloidosis, necessitating detailed serum analysis to exclude AL cardiac amyloidosis before making a final interpretation [5]. False negatives can also occur, particularly in patients with specific mutations, such as ATTRS77Y or Phe84Leu, underscoring the importance of cautious interpretation across all types of amyloidosis [[55], [56], [57]].

Radiotracer uptake is quantitatively assessed using the Perugini visual scoring scale, which rates cardiac uptake against skeletal uptake on a scale from 0 (no cardiac uptake) to 3 (cardiac uptake exceeding skeletal uptake). A score of 2 or higher indicates a positive scan, regardless of the radiotracer used [58]. This scoring system's accuracy largely depends on the interpreting professional's expertise, which can introduce variability in the assessment of amyloid burden [59,60]. Therefore, integrating SPECT/CT is recommended to improve diagnostic accuracy [60]. Furthermore, the reliability of cardiac scintigraphy findings can be influenced by external factors such as rib fractures, and valvular or annular calcifications, highlighting the importance of considering these elements in the diagnostic evaluation [61].

Positron Emission Tomography (PET) scanning serves as a valuable imaging method for diagnosing CA. This technique employs specific amyloid-binding radioactive tracers, including 11C-Pittsburgh B (11C-PiB) and 18F-florbetapir [[62], [63], [64]]. A prior meta-analysis comparing the diagnostic efficacy of 11C-PiB PET scans with CMR, and cardiac scintigraphy highlighted the potential of 11C-PiB PET scanning [65]. Yet, it revealed that cardiac scintigraphy offers superior diagnostic accuracy for identifying CA [65]. Owing to the limited recent PET studies on CA, PET scans were not included in this current meta-analysis. Nonetheless, advancements in PET imaging for the diagnosis of CA are anticipated in the future.

This meta-analysis demonstrates that both cardiac scintigraphy and CMR imaging are reliable methods for diagnosing CA. The choice of the most suitable diagnostic method should be customized based on the individual clinical scenario of each patient, considering factors such as cost-effectiveness and the availability of diagnostic modalities. This patient-centric strategy ensures that the strengths and weaknesses of each method are carefully weighed, leading to the most precise diagnosis and the highest quality of patient care.

For individuals with devices incompatible with CMR, as well as those suffering from renal insufficiency or claustrophobia, cardiac scintigraphy presents a more viable option. Conversely, CMR imaging is particularly beneficial for patients suspected of having AL amyloidosis or when the results from scintigraphy are inconclusive. The detailed myocardial visualization provided by CMR is crucial for distinguishing CA from other conditions with similar clinical presentations, such as hypertrophic cardiomyopathy. Furthermore, CMR is useful in differentiating between AL and TTR amyloidosis subtypes.

It is critical to acknowledge that while these imaging findings contribute significantly to the diagnostic process, they do not eliminate the need for histological confirmation and typing of amyloid in cases of CA. Therefore, integrating various diagnostic methods is recommended to enhance the accuracy of CA diagnoses wherever possible.

## 5 Limitations

A notable limitation of this analysis is the significant heterogeneity among the included studies. This heterogeneity likely stems from variations in patient demographics, imaging protocols, and reference standards across studies, which may influence the applicability of the findings across different patient populations. Additionally, the scarcity of prospective studies directly comparing non-invasive diagnostic techniques for CA represents a gap in the literature that challenges the definitive ranking of the diagnostic performance of cardiac scintigraphy versus CMR imaging. Addressing these challenges is crucial, and future research should focus on conducting well-designed, prospective studies that directly compare the diagnostic accuracy of these non-invasive techniques.

## 6 Conclusion

Cardiac scintigraphy, particularly with 99mTc-HMDP, offers superior diagnostic accuracy for CA compared to CMR. Controlled, randomized, prospective studies directly comparing these non-invasive techniques are essential to validate these findings. Future studies should also explore the role of emerging modalities like PET imaging in direct comparison with scintigraphy and CMR to further refine non-invasive diagnostic algorithms.

## Abbreviations

99mTc-DPD: Technetium-99 m-labelled 3,3-diphosphono-1,2-propanodicarboxylic acid scintigraphy
99mTc-HMDP: Technetium-99 m-labelled hydroxymethylene- diphosphonate scintigraphy
99mTc-PYP: 99mTechnetium-pyrophosphate scintigraphy
AL amyloidosis: amyloid light chain amyloidosis
CA: Cardiac amyloidosis
CMR: Cardiac Magnetic Resonance
dOR: diagnostic odds ratio
NLR: negative likelihood ratio
PLR: positive likelihood ratio
QUADAS-2: A Revised Tool for the Quality Assessment of Diagnostic Accuracy Studies
ROC: receiver operating characteristic curve
TTR amyloidosis: Transthyretin amyloidosis

## CRediT authorship contribution statement

**Mahmoud Balata:** Writing – original draft, Methodology, Funding acquisition, Data curation, Conceptualization. **Abdelrahman M.Attia:** Software, Resources, Formal analysis. **Mohamed Ibrahim Gbreel:** Software, Resources, Methodology, Formal analysis. **Mamdouh Elsmaan:** Software, Methodology, Investigation. **Marwa Hassan:** Writing – review & editing, Supervision. **Paul Rapeanu:** Supervision. **Milka Marinova:** Supervision, Methodology, Formal analysis. **Marc Ulrich Becher:** Validation, Supervision. **Islam Ebeid:** Writing – review & editing. **Jasmin Ortak:** Supervision. **Hüseyin Ince:** Writing – review & editing, Visualization, Validation, Conceptualization.

## Consent

The authors have nothing to report.

## Ethics statement

The authors have nothing to report.

## Funding

No funding was received for this work.

## Declaration of competing interest

The authors declare that they have no known competing financial interests or personal relationships that could have appeared to influence the work reported in this paper.

## Data Availability

Most of the data generated or analyzed during this study are included in the supplementary information files. All additional datasets used or analyzed during the current study are available from the corresponding author on reasonable request.

## Appendix A Supplementary data

Supplementary material Image 1
