# Supplementary material for: Cardiac magnetic resonance imaging and cardiac scintigraphy in the diagnosis of cardiac amyloidosis: A meta-analysis of 4866 patients
Source: J Mol Cell Cardiol Plus. 2025 Oct 17;14:100489. doi: 10.1016/j.jmccpl.2025.100489 (PMC12557567; doi:10.1016/j.jmccpl.2025.100489)
Supplement: Supplementary file 1 — Supplementary material [file mmc1.docx]

**Supplemental material**

# Supplemental Table 1: Baseline patient characteristics of cardiac scintigraphy studies.

| **Study ID** | **Study** design | **Number** | **Population** | **Age, mean (SD)** | **Reference test** | **Index test** | **Radiotracer** | **Uptake time, hours** | **Scintigraphic image** | **Image analysis** |
| --- | --- | --- | --- | --- | --- | --- | --- | --- | --- | --- |
| Bokhari 2013 | Prospective cohort | 45 | AL 12 (26.6) ATTRwt 16 (35.6) ATTRm 17 (37.8) | 70 (2) | Biopsy/ one involved organ with echocardiographically evidence of amyloid CM | Nuclear Scintigraphy | 99mTc-PYP | 1.13 | Planar (WB) and SPECT (TH) | Qualitative and semi-quantitative |
| Capelli 2017 | Retrospective cohort | 131 | AL 26 (40%) TTRm 16 (24.6%) TTRwt 23 (35.4) LVH 66 (50.4) | 79 (8) | Biopsy or clinical criteria | Nuclear Scintigraphy | 99mTc-HMDP | 2.5 | Planar (WB) | Qualitative and semi-quantitative |
| Cariou 2017 | Prospective cohort | 114 | TTRm 5 (4.4%) TTRwt 26 (22.8%) AA 1 (0.8%) AL 18 (15.8%) HCM 19 (16.7%) Unspecified 45 (39.5%) | 72 (12) | Biopsy or clinical criteria | Nuclear Scintigraphy | 99mTc-DPD | NA | Planar (WB) | Semi-quantitative |
| Castano 2016 | Retrospective cohort | 171 | AL 34 (19.9%) TTRun 12 (7.01%) TTRm 37 (21.6%) non-Amyloid 16 (9.4%) TTRwt 72 (42.1%) | 73(65–79) | Biopsy or clinical criteria | Nuclear Scintigraphy | 99mTc-PYP | 1 to 3 | Planar (TH) | Qualitative and semi-quantitative |
| Flaherty 2019 | Prospective cohort | 43 | AL 3 (6.9%) TTRun 1 (2.3%) TTRm 3 (6.9%) TTRwt 20 (46.5%) non-Amyloid 16 (37.2%) | 77 (8) | Biopsy or clinical criteria | Nuclear Scintigraphy | 99mTc-PYP | 1 | Planar (WB) | Qualitative and quantitative |
| Gallini 2019 | Retrospective cohort | 76 | AL 12 TTRm 16  TTRwt 37 LVH 11 | 78 (7) | Biopsy or clinical criteria | Nuclear Scintigraphy | 99mTc-HMDP | 2.5 | Planar (WB) and SPECT | Qualitative and semi-quantitative |
| Gilmore 2016 | Retrospective cohort | 1217 | Non cardic 499 (41%) cardiac 718 (59%) | 62 (34-76) | Biopsy or clinical criteria | Nuclear Scintigraphy | 99mTc-DPD 99mTc-PYP 99mTc-HMDP | 1 to 3 | Planar (WB) | Qualitative and semi-quantitative |
| Kessler 2023 | Retrospective cohort | 136 | AL 5 (3.7%) TTRm 5 (3.7%) TTRwt 38 (28%) non-Amyloid 88 (64.7%) | 76 (10) | Biopsy or clinical criteria | Nuclear Scintigraphy | 99mTc-DPD | 3 | SPECT/CT (TH) | Semi-quantitative |
| Masri 2020 | Prospective cohort | 233 | TTRm 8 (3.4%) TTRwt 52 (22.3%) Other 173 (74.3%) | 77(69–83) | Biopsy or clinical criteria | Nuclear Scintigraphy | 99mTc-PYP | 3 | Planar (WB) and SPECT/CT (TH) | Semi-quantitative |
| Matsuda 2023 | Retrospective cohort | 32 | ATTR 8 (25%) non-ATTR 24 (75%) | 76(69–83) | Biopsy or clinical criteria | Nuclear Scintigraphy | 99mTc-PYP | 1 | SPECT/CT (TH) | Semi-quantitative |
| Moore 2017 | Prospective cohort | 21 | AL 8 (38.1%) TTRm 3 (14.3%) TTRwt 8 (38.1%) Other 2 (9.5%) | 70 | Biopsy or clinical criteria | Nuclear Scintigraphy | 99mTc-DPD | 3 | Planar (WB) and SPECT/CT (TH) | Qualitative and semi-quantitative |
| Moral 2012 | Retrospective cohort | 19 | AL 11 (57.9%) TTRm 3 (15.8%) TTRwt 5 (26.3%) | 64 (15) | Biopsy or clinical criteria | Nuclear Scintigraphy | 99mTc-DPD | 3 | Planar (WB) and SPECT/CT (TH) | Qualitative |
| Papantoniou 2015 | Retrospective cohort | 12 | AL 6 (50%) TTR 6 (50%) | 70.6 (13.2) | Biopsy or clinical criteria | Nuclear Scintigraphy | 99mTc-PYP | 1,2, or3 | Planar (WB) and SPECT (TH) | Qualitative and semi-quantitative |
| Poterucha 2021 | Retrospective cohort | 753 | TTRwt 196 (26%) TTRm 18 (2%) No Amyloidosis 456 (61%) Other 83 (11%) | 77 (11) | Biopsy or clinical criteria | Nuclear Scintigraphy | 99mTc-PYP | 1 | Planar (WB) and SPECT (TH) | Qualitative |
| Rapezzi 2011 | Retrospective cohort | 94 | AL 34 (36.2%) TTRm 28 (29.7%) TTRwt 17 (18.1) Other 15 (16%) | 62 (14) | Biopsy or clinical criteria | Nuclear Scintigraphy | 99mTc-DPD | 3.08 | Planar (WB) and SPECT (TH) | Qualitative and semi-quantitative |
| Sperry 2020 | Retrospective cohort | 109 | TTRwt 33 (30.2%) Other 76 (69.7%) | 77(72–82) | Biopsy or clinical criteria | Nuclear Scintigraphy | 99mTc-PYP | 3 | Planar (WB) and SPECT (TH) | Semiquantitative |
| 99mTc-PYP, 99mTc-pyrophosphate; 99mTc-HMDP, 99mTc-hydroxymethylene diphosphonate; 99mTc-DPD, 99mTc-3,3-diphosphono-1,2-propanodicarboxylic acid; AL, amyloid light-chain; CA, cardiac amyloidosis; LV, left ventricular; NA, not available; Other, refers to other type of cardiac amyloidosis or non-amyloidosis; TH, thorax; TTR, transthyretin; TTRm, mutant TTR; TTRwt, wildtype TTR; TTRun, unknown genotype TTR; WB, whole body. | | | | | | | | | | |

# Supplemental Table 2: Baseline patient characteristics of cardiac magnetic resonance (CMR) studies.

| **Study ID** | **Study design** | **Study population** | **No. patients** | **Age** | **Reference test** | **Index test** | **Image analysis** |
| --- | --- | --- | --- | --- | --- | --- | --- |
| Austin 2009 | Retrospective cohort | Suspected CA | 47 | 62 (51 to 75) | Biopsy from any organ | LGE sub endocardium | Quantitative assessment |
| Baroni 2018 | Retrospective cohort | Suspected CA | 21 | 57 (13) | Myocardial biopsy | LGE sub endocardium | Semi-quantitative |
| Bhatti 2016 | Retrospective cohort | Plasma cell dyscrasia | 42 | 63 (56-69) | Myocardial biopsy | LGE (any) | Quantitative assessment |
| Damy 2015 | Retrospective cross-sectional | Suspected CA | 298 | 62 (50-74) | Myocardial biopsy | LGE (any) | Qualitative assessment |
| Dungu 2014 | Retrospective cohort | Proved CA | 97 | 68 (9) | Biopsy from any organ | LGE sub endocardium | Semi-quantitative |
| Fontana 2015 | Prospective cohort | Proved CA | 250 | 67 (12) | Biopsy from any organ | LGE (any) | NA |
| Gregorio 2016 | Prospective cohort | Proved CA | 32 | 58 (11) | Biopsy from any organ | LGE LV | Qualitative assessment |
| Hosch 2007 | Prospective cohort | Systemic amyloidosis | 29 | 59 (6.1) | Biopsy from any organ | Native T1 | NA |
| Karamitsos 2013 | Retrospective cohort | Systemic amyloidosis | 42 | 63 (9) | Biopsy from any organ | LGE (any) | Quantitative assessment |
| Kristen 2015 | Prospective cohort | Proved CA | 125 | 62.3 (12.9) | Biopsy from any organ | LGE RV | Quantitative assessment |
| Kwong 2015 | Prospective cohort | Proved CA | 81 | 63 (17) | Myocardial biopsy | LGE atria | Quantitative assessment |
| Maceira 2005 | Prospective cohort | Proved CA | 45 | 60 (11) | Biopsy from any organ | LGE sub endocardium | Quantitative assessment |
| Mongeon 2012 | Retrospective cross-sectional case-control study | Proved CA | 38 | 61 (12) | Myocardial biopsy | LGE LV | Qualitative assessment |
| Naharro 2017 | Prospective cohort | Proved CA | 313 | 72 (11) | Biopsy from any organ | LGE transmural | NA |
| Ruberg 2009 | Prospective cohort | Systemic amyloidosis | 28 | 62 (11) | Biopsy from any organ | LGE (any) | NA |
| Syed 2010 | Retrospective cohort | Proved CA | 35 | 60 (11) | Myocardial biopsy | LGE sub endocardium | Semi-quantitative |
| Ternacle 2016 | Prospective cohort | Proved CA | 79 | 71 (13) | Biopsy from any organ | LGE LV | Quantitative assessment |
| Vogelsberg 2008 | Prospective cohort | Suspected CA | 33 | 64 (13) | Myocardial biopsy | LGE sub endocardium | NA |
| White 2014 | Prospective cohort | Suspected CA | 25 | 60 (13) | Myocardial biopsy | LGE LV | Quantitative assessment |
| CA, cardiac amyloidosis; LGE, Late Gadolinium Enhancement; LV, left ventricular; NA, not available. | | | | | | | |

# Supplemental Table 3: Assessment of risk of bias and applicability using QUADAS-2 tool:

| Study ID | Risk of bias | | | | Applicability concerns | | |
| --- | --- | --- | --- | --- | --- | --- | --- |
|  | Patient selection | Index test | Reference standard | Flow and timing | Patient selection | Index test | Reference standard |
| Austin 2009 | 2 | 0 | 0 | 2 | 0 | 0 | 0 |
| Baroni 2018 | 2 | 0 | 0 | 2 | 0 | 0 | 0 |
| Bhatti 2016 | 2 | 0 | 0 | 2 | 0 | 0 | 0 |
| Bokhari 2013 | 2 | 0 | 0 | 0 | 0 | 0 | 0 |
| Capelli 2017 | 1 | 0 | 0 | 0 | 2 | 0 | 0 |
| Cariou 2017 | 2 | 0 | 0 | 2 | 0 | 0 | 0 |
| Castano 2016 | 1 | 2 | 0 | 0 | 2 | 0 | 0 |
| Damy 2015 | 0 | 0 | 0 | 0 | 2 | 0 | 0 |
| Dungu et 2014 | 0 | 0 | 0 | 2 | 0 | 0 | 0 |
| Flaherty 2019 | 0 | 0 | 0 | 0 | 0 | 0 | 0 |
| Fontana 2015 | 0 | 0 | 0 | 0 | 0 | 0 | 0 |
| Gallini 2019 | 2 | 1 | 0 | 0 | 2 | 0 | 0 |
| Gilmore 2016 | 2 | 1 | 0 | 0 | 2 | 0 | 0 |
| Gregorio 2016 | 0 | 0 | 0 | 2 | 0 | 0 | 0 |
| Hosch 2007 | 0 | 0 | 0 | 2 | 0 | 0 | 0 |
| Karamitsos 2013 | 0 | 0 | 0 | 0 | 0 | 0 | 0 |
| Kessler 2023 | 0 | 0 | 1 | 0 | 0 | 0 | 0 |
| Kristen 2015 | 0 | 0 | 0 | 2 | 0 | 0 | 0 |
| Kwong 2015 | 0 | 0 | 0 | 0 | 0 | 0 | 0 |
| Maceira 2005 | 0 | 0 | 0 | 2 | 0 | 0 | 0 |
| Masri 2020 | 0 | 0 | 1 | 0 | 0 | 0 | 0 |
| Matsuda 2023 | 1 | 0 | 1 | 0 | 0 | 0 | 0 |
| Mongeon 2012 | 0 | 0 | 0 | 0 | 0 | 0 | 0 |
| Moore 2017 | 0 | 0 | 0 | 0 | 0 | 0 | 0 |
| Moral 2012 | 1 | 2 | 0 | 0 | 0 | 0 | 0 |
| Naharro 2017 | 0 | 0 | 0 | 2 | 0 | 0 | 0 |
| Papantoniou 2015 | 1 | 0 | 1 | 0 | 1 | 0 | 1 |
| Poterucha 2021 | 1 | 1 | 1 | 0 | 0 | 0 | 0 |
| Rapezzi 2011 | 0 | 0 | 0 | 0 | 2 | 0 | 0 |
| Ruberg 2009 | 0 | 0 | 0 | 2 | 0 | 0 | 0 |
| Sperry 2020 | 0 | 0 | 1 | 0 | 0 | 0 | 0 |
| Syed 2010 | 0 | 0 | 0 | 0 | 0 | 0 | 0 |
| Ternacle 2016 | 0 | 0 | 0 | 2 | 0 | 0 | 0 |
| Vogelsberg 2008 | 2 | 0 | 0 | 0 | 0 | 0 | 0 |
| White 2014 | 2 | 0 | 0 | 2 | 0 | 0 | 0 |
| Risk of bias assessment. 0, low risk; 1, unclear risk; 2, high risk. | | | | | | | |

**Supplemental Figure 1**: The dOR and ROC curve analysis of 99mTc-DPD Imaging:


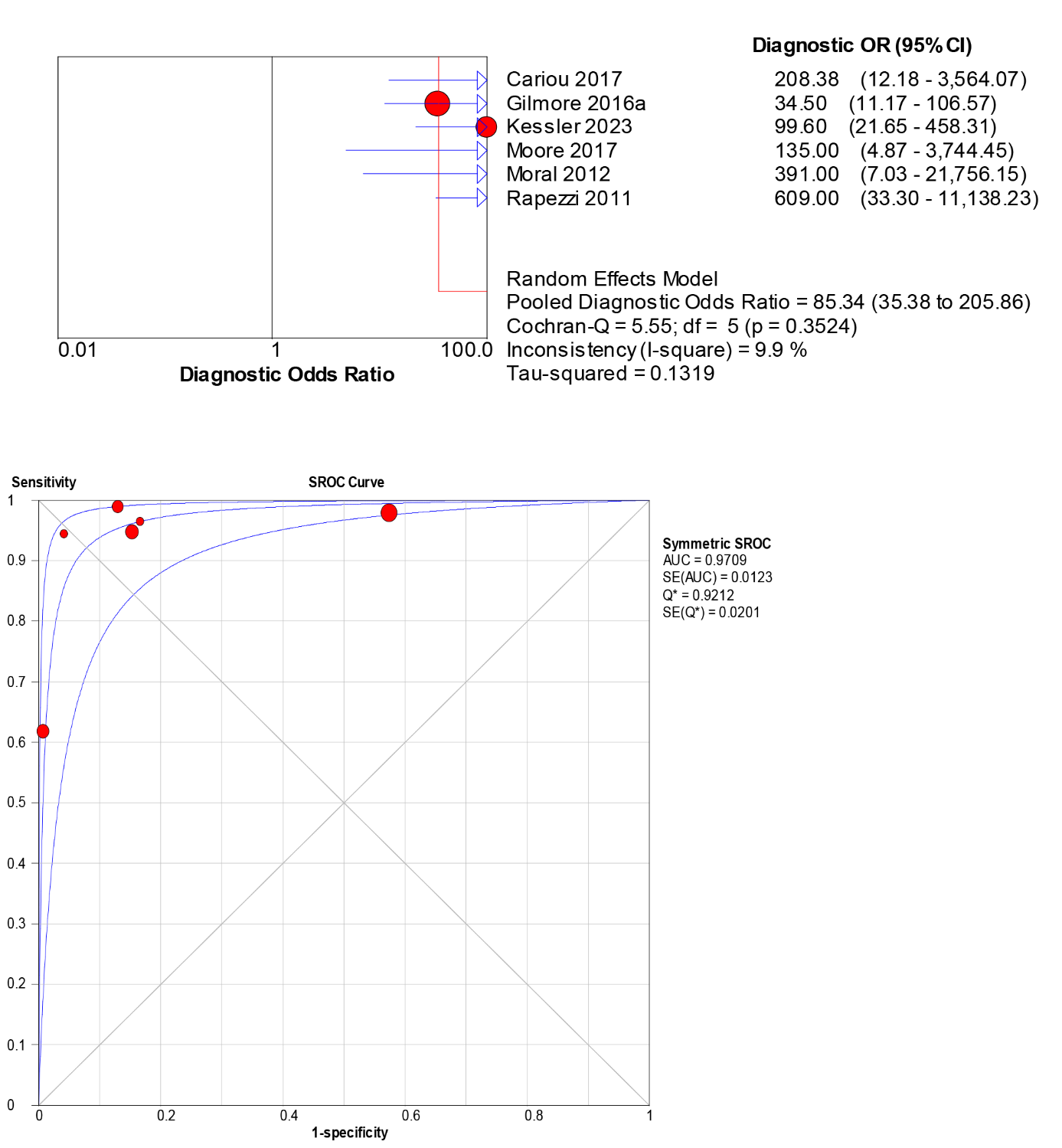


Pooled dOR = 85.34 (95% CI: 35.38–205.86); AUC = 0.97. Error bars represent 95% CI. Heterogeneity: I² = 9.9%. Model: random-effects. (see Supplementary Table 1 for study-level counts)

**Supplemental Figure 2**: The dOR and ROC curve analysis of 99mTc-HMDP Imaging:


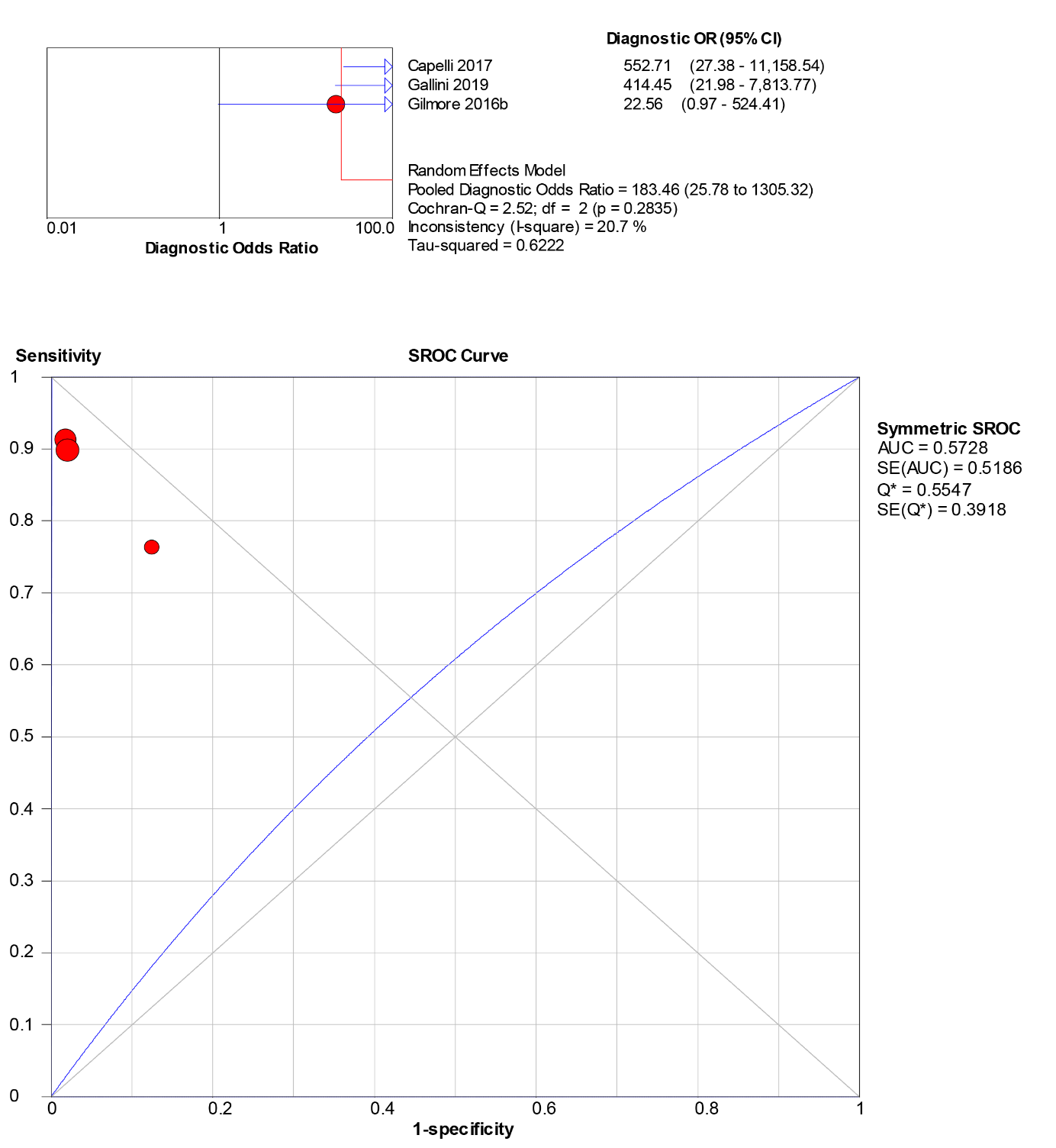


Pooled dOR = 183.46 (95% CI: 25.78–1305.32); AUC = 0.57. Error bars represent 95% CI. Heterogeneity: I² = 20.7%. Model: random-effects. (see Supplementary Table 1 for study-level counts)

**Supplemental Figure 3**: The dOR and ROC curve analysis of 99mTc-PYP Imaging


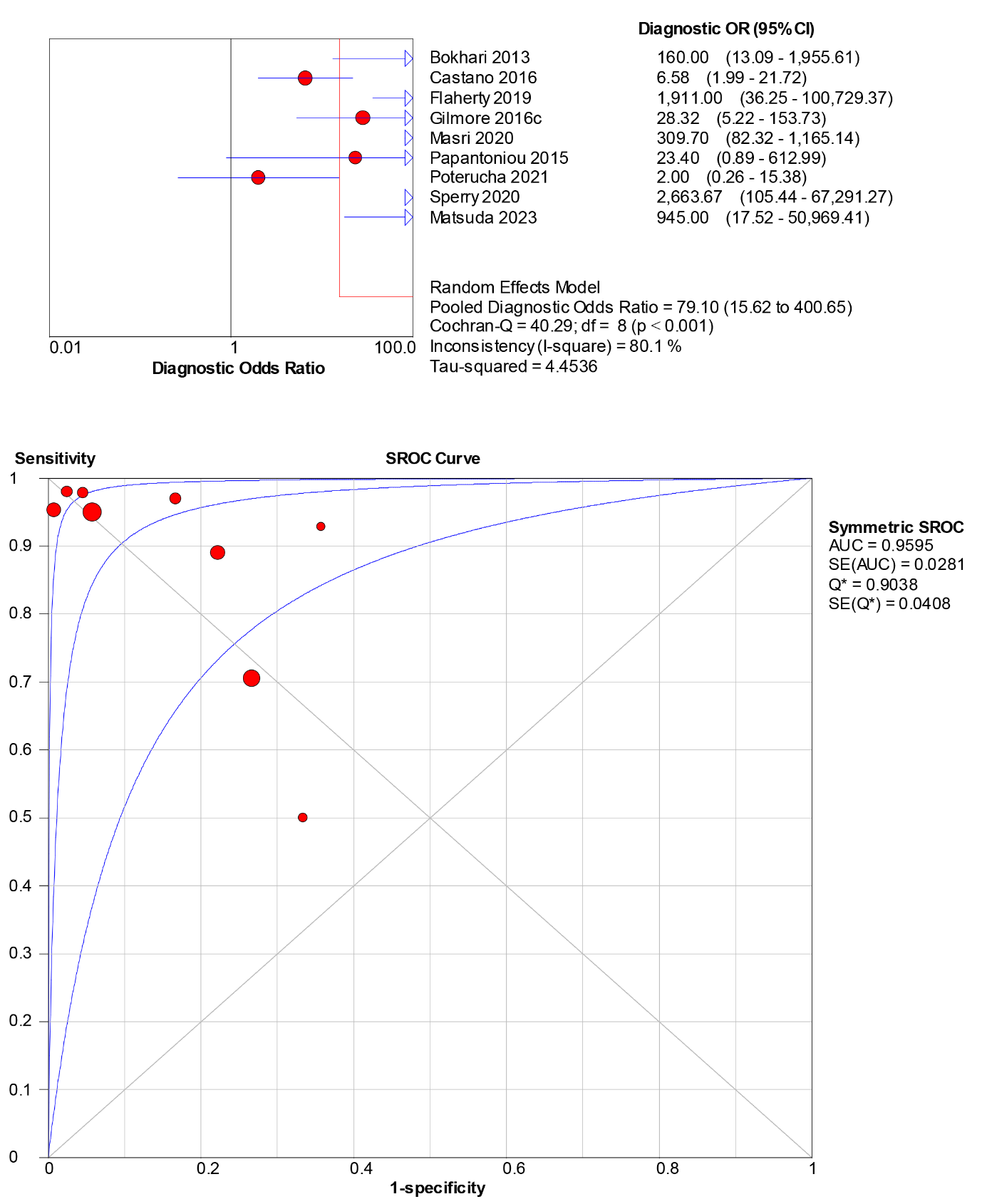


Pooled dOR = 79.10 (95% CI: 15.62–400.65); AUC = 0.96. Error bars represent 95% CI. Heterogeneity: I² = 80.1%. Model: random-effects. (see Supplementary Table 1 for study-level counts)

**Supplemental Figure 4**: The general dOR and ROC curve analysis of cardiac scintigraphy:


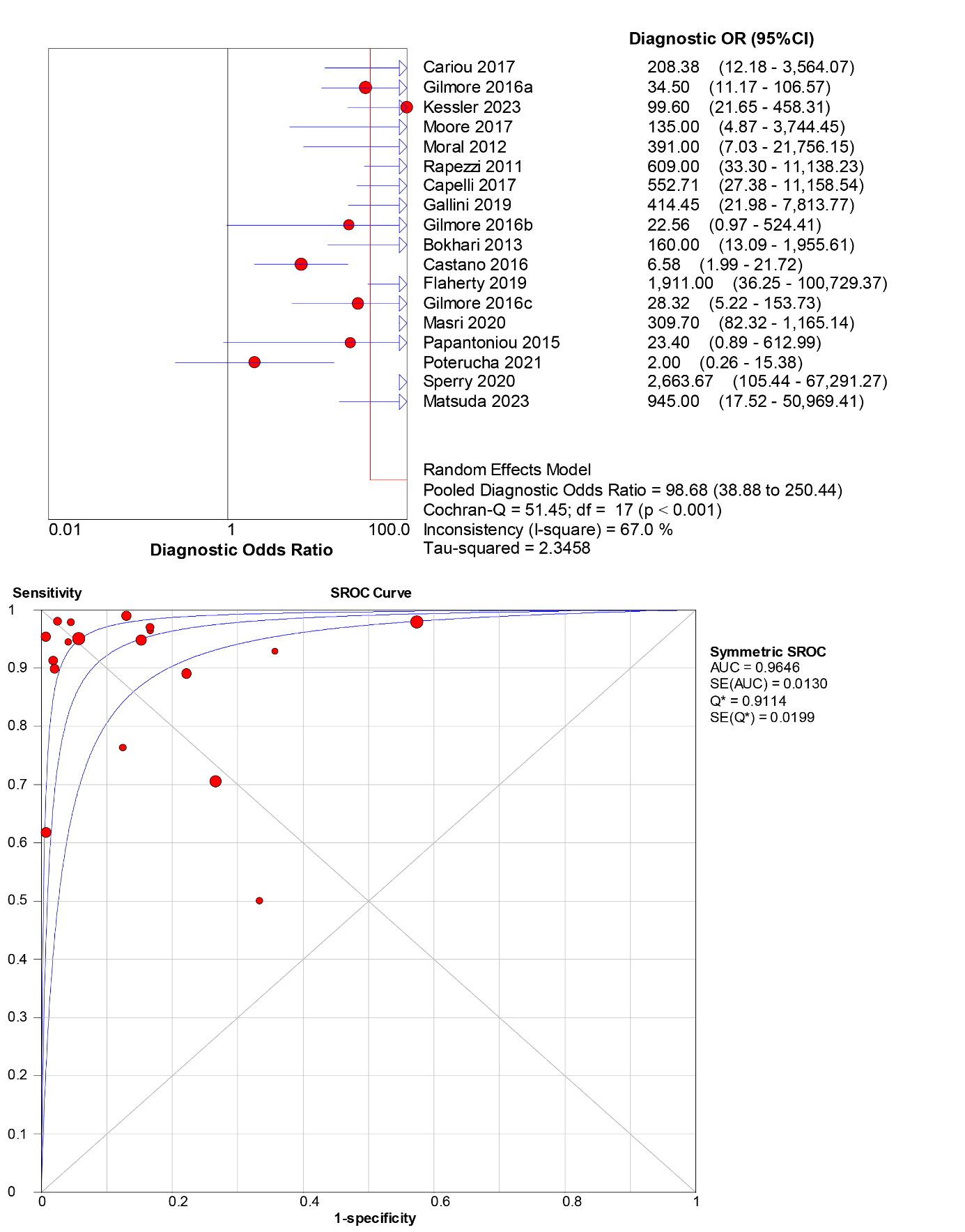


Pooled dOR = 98.68 (95% CI: 38.88–250.44); AUC = 0.96. Error bars represent 95% CI. Heterogeneity: I² = 67.0%. Model: random-effects. (see Supplementary Table 1 for study-level counts)

**Supplemental Figure 5**: The dOR and ROC curve analysis of CMR:


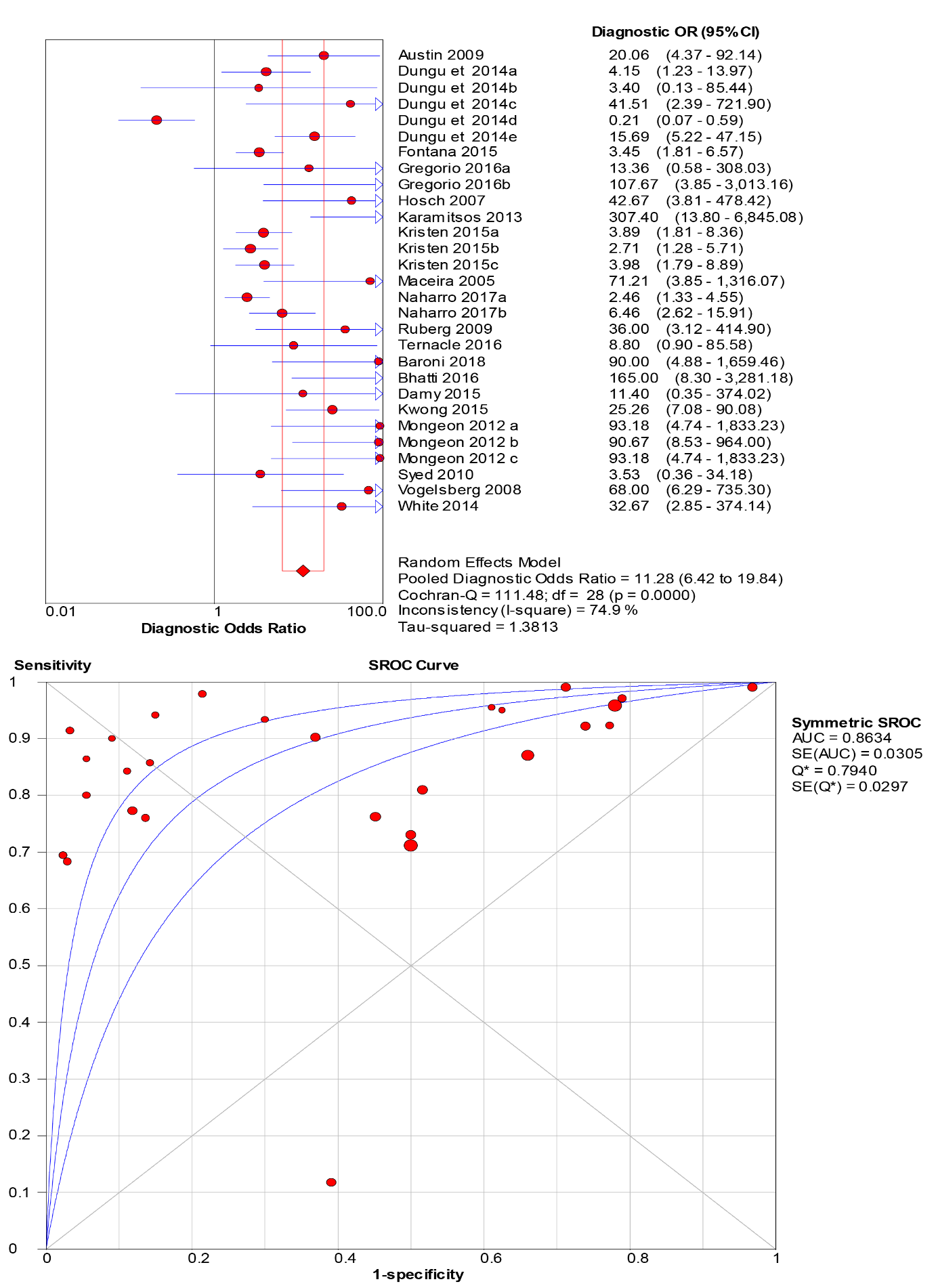


Pooled dOR = 11.28 (95% CI: 6.42–19.84); AUC = 0.86. Error bars represent 95% CI. Heterogeneity: I² = 74.9%. Model: random-effects. (see Supplementary Table 2 for study-level counts)
